# Paying attention to smell: cholinergic signaling in the olfactory bulb

**DOI:** 10.3389/fnsyn.2014.00021

**Published:** 2014-09-25

**Authors:** Rinaldo D. D’Souza, Sukumar Vijayaraghavan

**Affiliations:** Department of Physiology and Biophysics and the Neuroscience Program, School of Medicine, University of ColoradoAurora, CO, USA

**Keywords:** muscarinic, nicotinic, glomerular, GABAergic, filter

## Abstract

The tractable, layered architecture of the olfactory bulb (OB), and its function as a relay between odor input and higher cortical processing, makes it an attractive model to study how sensory information is processed at a synaptic and circuit level. The OB is also the recipient of strong neuromodulatory inputs, chief among them being the central cholinergic system. Cholinergic axons from the basal forebrain modulate the activity of various cells and synapses within the OB, particularly the numerous dendrodendritic synapses, resulting in highly variable responses of OB neurons to odor input that is dependent upon the behavioral state of the animal. Behavioral, electrophysiological, anatomical, and computational studies examining the function of muscarinic and nicotinic cholinergic receptors expressed in the OB have provided valuable insights into the role of acetylcholine (ACh) in regulating its function. We here review various studies examining the modulation of OB function by cholinergic fibers and their target receptors, and provide putative models describing the role that cholinergic receptor activation might play in the encoding of odor information.

## INTRODUCTION

The network of cholinergic fibers acts as a major neuromodulatory system in the brain. It is not only implicated in the pathophysiology of neurodegenerative disorders like Alzheimer’s disease, but it also plays a central role in the functioning of the healthy brain. The release of ACh by these fibers is involved in the enhancement of sensory perception during wakefulness, particularly during periods of sustained attention (Himmelheber et al., 2000; Jones, 2005). Studying how ACh controls various brain systems at the level of circuits and synaptic transmission is critical for the understanding of how ACh affects brain function, both in health and in disease. The mammalian main OB provides for a convenient model system to study the modulatory control of sensory circuits. It is located centrally in the olfactory pathway (only one synapse away from odor input into the nose and one synapse away from higher cortical processing), and its excitatory and inhibitory neurons are relatively well-segregated. Importantly, its circuits and function are strongly modulated by ACh. Cholinergic input to the OB is provided primarily by axons of neurons whose cell bodies reside in the HDB in the basal forebrain (Wenk et al., 1980; Senut et al., 1989). While a more recent study has demonstrated the presence of choline acetyltransferase (ChAT)-expressing neurons within the OB itself (Krosnowski et al., 2012), a functional role has yet to be ascribed to these cholinergic interneurons. ACh released by HDB cholinergic neurons acts on both, nicotinic and muscarinic receptors (nAChR and mAChR, respectively) resulting in the control of olfactory function that is dependent upon the brain state of the animal – whether it is sleeping, performing a task, or simply awake and immobile. In this review, we focus on studies that have helped us gain better insights into how the release of ACh in the OB affects olfaction at the cellular, circuit, and behavioral level, and discuss how it might modulate odor coding during attentional control of OB circuits.

## MULTIPLE, COMPLEX MECHANISMS INVOLVED IN OLFACTORY CODING

**FIGURE 1:**
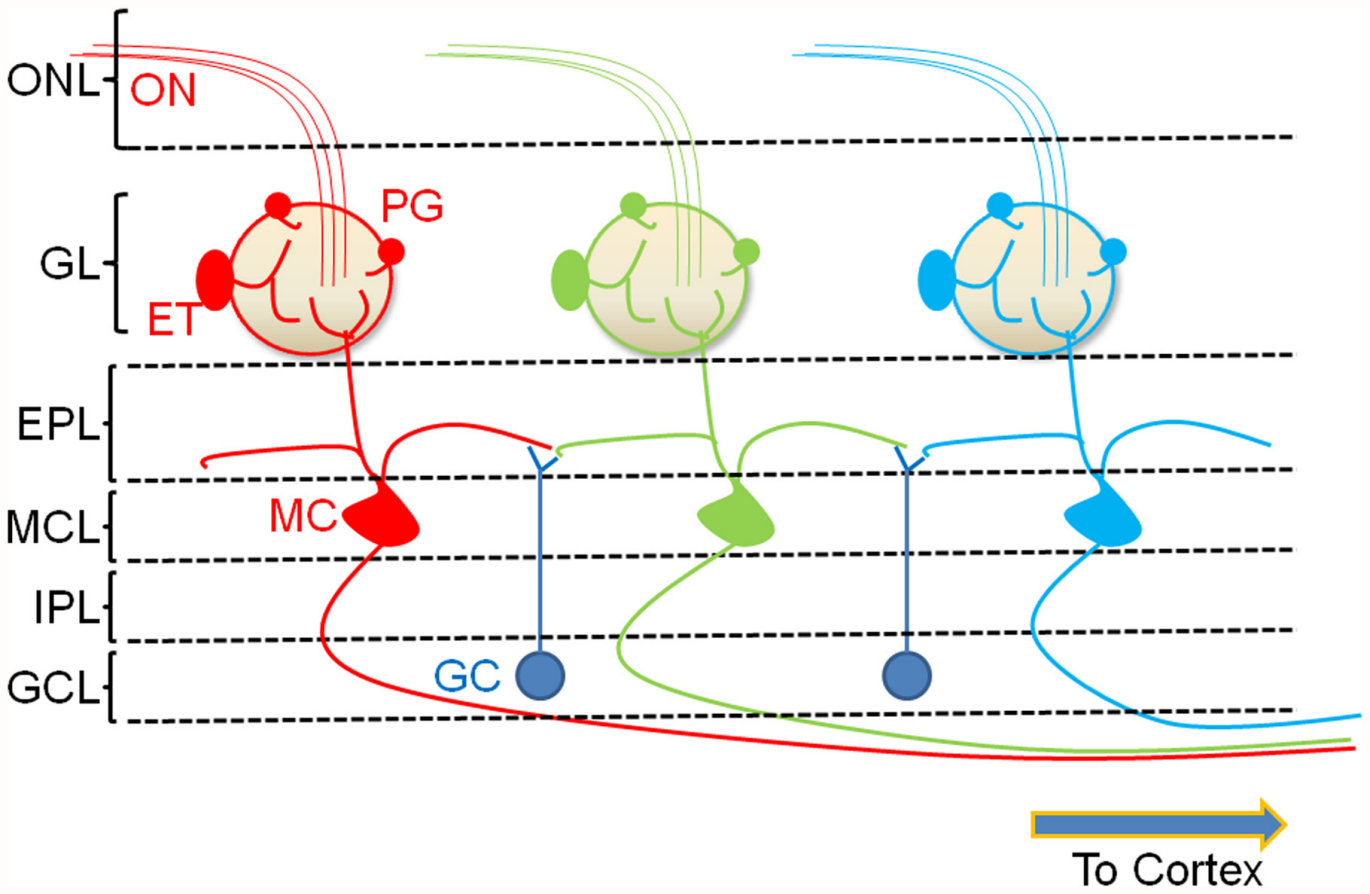
**A simplified cartoon of the bulbar circuit. ORNs in the nasal epithelium send their axons ON to the glomerular layer of the OB.** Each glomerulus receives convergent ON input from ORNs that recognize the same odor epitope (color coded). Resident interneurons in the juxtaglomerular region receive ON input and modulate glomerular signaling. The two major glomerular interneuron classes are the glutamatergic external tufted (ET) cells and the GABAergic PG cells. Together, these cells regulate the glomerular output resulting in the excitation of MCs, the principal neurons of the OB. A second interneuron type, the granule cell (GC), forms dendrodendritic synapses with the lateral dendrites of the MCs. These neurons exert GABAergic control over multiple MCs resulting in lateral inhibition. The modulated output of the MC is then transmitted to the pyriform cortex. ONL, olfactory nerve layer; GL, glomerular layer; EPL, external plexiform layer; MCL, mitral cell layer; IPL, internal plexiform layer; GCL, granule cell layer.

The OB represents a convergence point for incoming odor signals and contains the synapse transferring odor information between the ORNs and higher cortical regions. ORNs send their axons (which form the ON) into defined structures called glomeruli (**Figure 1**). Projections from ORNs that recognize the same odor epitope converge onto about two (of about two thousand) glomeruli in the ipsilateral bulb (Vassar et al., 1994). Within the glomerular neuropil, these neurons provide direct (Najac et al., 2011) and indirect (Najac et al., 2011; Gire et al., 2012) synaptic inputs onto the MCs, the principal output neurons of the OB. Modulation of odor information provided by these inputs occurs in the glomerulus as well as in the other layers of the bulb by a number of bulbar interneurons. Two key neuronal cell types that modulate glomerular output are the GABAergic PG cells and the glutamatergic external tufted (ET) cells, both of which are also directly excited by ON input (Gire and Schoppa, 2009). The lateral dendrites of the MCs receive a second set of GABAergic inputs from granule cells (GCs) within another distinct layer called the external plexiform layer (EPL; **Figure 1**). Other interneuron types and subtypes have been described (Batista-Brito et al., 2008) but are not considered here in the context of cholinergic modulation.

Much of the information on odor representations (Xu et al., 2000; Bozza et al., 2004) and MCs responses to odor (Kashiwadani et al., 1999) comes from studies on anesthetized animals. However, recent studies have shown a much more complex scenario in awake animals, requiring re-evaluation of our notions of olfactory processing (Kato et al., 2012; Wachowiak et al., 2013). MCs in awake, behaving animals are spontaneously active (Rinberg and Gelperin, 2006; Rinberg et al., 2006; Davison and Katz, 2007) with firing that is often locked to the respiration cycle (Cury and Uchida, 2010; Wachowiak, 2011). Odor-evoked responses are not encoded in simple changes in firing frequencies; instead, the OB adopts various sophisticated mechanisms, involving the activity of MCs, to detect and encode odors. For example, upon odor onset, the latency of the first MC spike in response to the odor (Margrie and Schaefer, 2003; Junek et al., 2010), reduction in MC firing frequency (Rinberg and Gelperin, 2006; Rinberg et al., 2006; Davison and Katz, 2007), alterations in the relative temporal phase of individual spikes (Dhawale et al., 2010), relative timing of MC spikes (Haddad et al., 2013), and fine-scale changes in temporal spike patterns (Friedrich and Laurent, 2001; Cury and Uchida, 2010) are all thought to play important roles in odor coding. Each of these mechanisms is a potential target for modulation, thus leading to a multifold increase in the computational power of the OB.

It has now been demonstrated that the OB is not merely an encoder of odor information that is subsequently decoded downstream in the cortex, but that it is itself involved in “higher order” processing. The response of MCs to odors, for example, depends not only on the chemical structure of odorant molecules, but also on more behaviorally relevant properties. *In vivo* recordings have shown that synchrony between MC spiking, in response to an odor, can be altered depending on whether the odor is rewarded in a behavioral task or not (Doucette and Restrepo, 2008; Doucette et al., 2011). Such an associative cortex-like feature (Doucette et al., 2011) suggests an advanced role for the OB in sensory information processing. This is consistent with studies which show that the activity of OB neurons can be profoundly affected by feedback inputs from the cortex (Gao and Strowbridge, 2009; Markopoulos et al., 2012). Task-dependent control of circuits in the OB thus plays a vital role in processing odor information.

## THE OLFACTORY BULB AND ITS CHOLINERGIC INPUT

A cluster of cholinergic neurons from the basal forebrain sends diffuse projections to the entire cortical mantle. All cortical areas receive cholinergic innervation, though there appears to be differences in the density of innervation across specific layers (Lysakowski et al., 1989; Mesulam et al., 1992). The lack of consistent topographic precision leads to the idea that cholinergic activation might lead to uniform effects across structures. However, there are different clusters of basal forebrain cholinergic neurons that have been identified and described that might suggest modality-specific control by the transmitter (Zaborszky, 2002).

The cholinergic input from the HDB is a major centrifugal projection into the OB. Cholinergic neurons of the basal forebrain regulate cortical activity in a state-dependent manner. These neurons fire bursts of action potentials during awake and paradoxical sleep states while remaining more or less silent during slow wave sleep (Jones, 2004, 2005). During active periods, the burst discharge of these neurons appears to be synchronized with gamma and theta oscillations (Lee et al., 2005).

**FIGURE 2:**
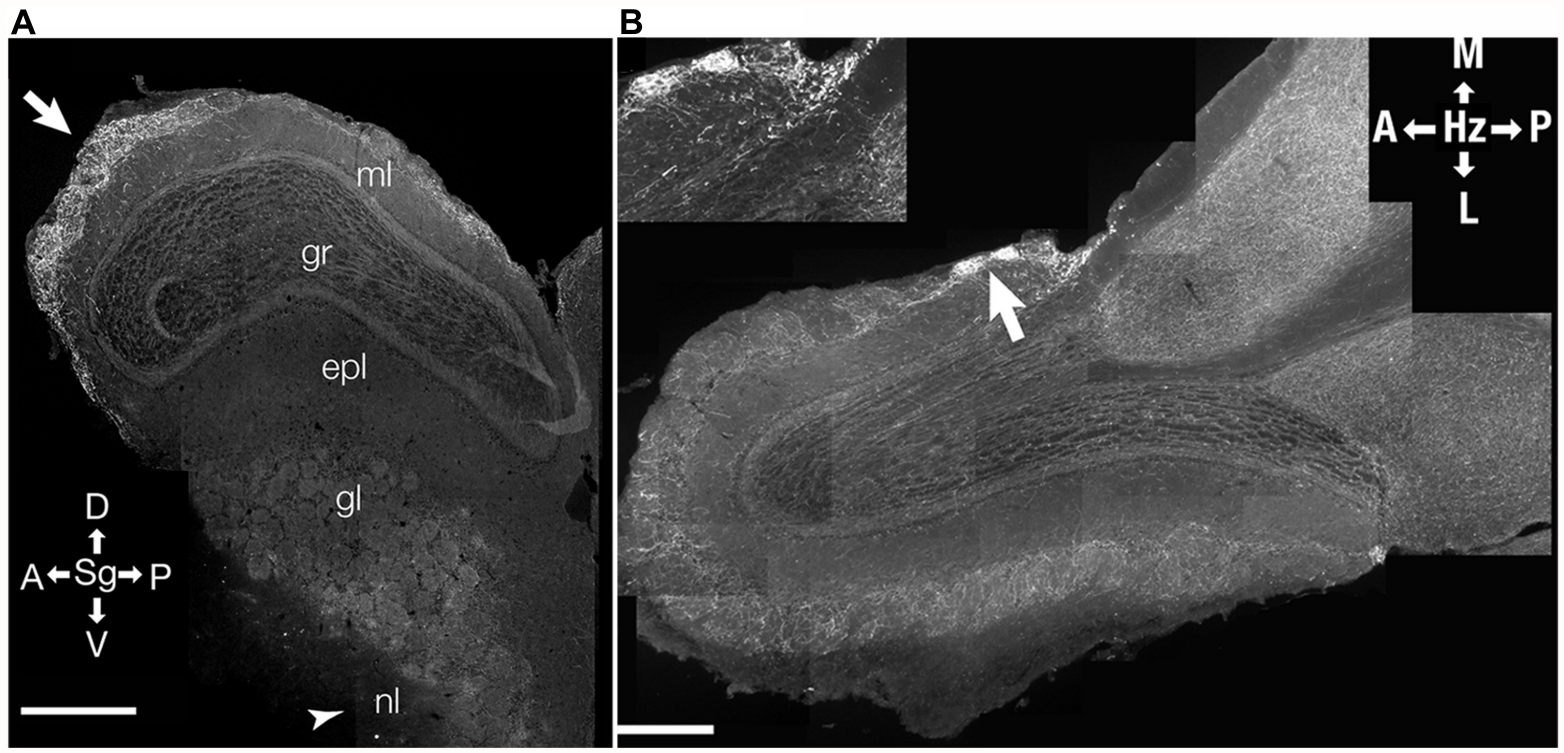
**Distribution of cholinergic innervation in the OB.** Distribution of incoming cholinergic fibers from the HDB was examined in sections from a 3 month-old mouse expressing a tauGFP fusion protein under a choline acetyltransferase promoter (ChAT-tauGFP mouse). **(A)** Parasagittal section (Sg). *Arrow* points to region of relatively heavy GFP labeling in the anterior glomerular region of the bulb. *Arrow head* indicates the olfactory nerve layer (nl) where relatively little labeling is found. ml- mitral cell layer; gr, granule cell layer; epl, external plexiform layer; gl, glomerular layer; nl, olfactory nerve layer. **(B)** Micrograph of a horizontal (Hz) cross-section of the OB. Arrow points to heavily stained atypical glomeruli shown in inset. **(B** inset**)** High-resolution micrograph of two atypical glomeruli with a relatively high amount of GFP staining. D, dorsal; V, ventral; A, anterior; P, posterior; L, lateral; M, medial. Data, with permission, from Salcedo et al. (2011).

**FIGURE 3:**
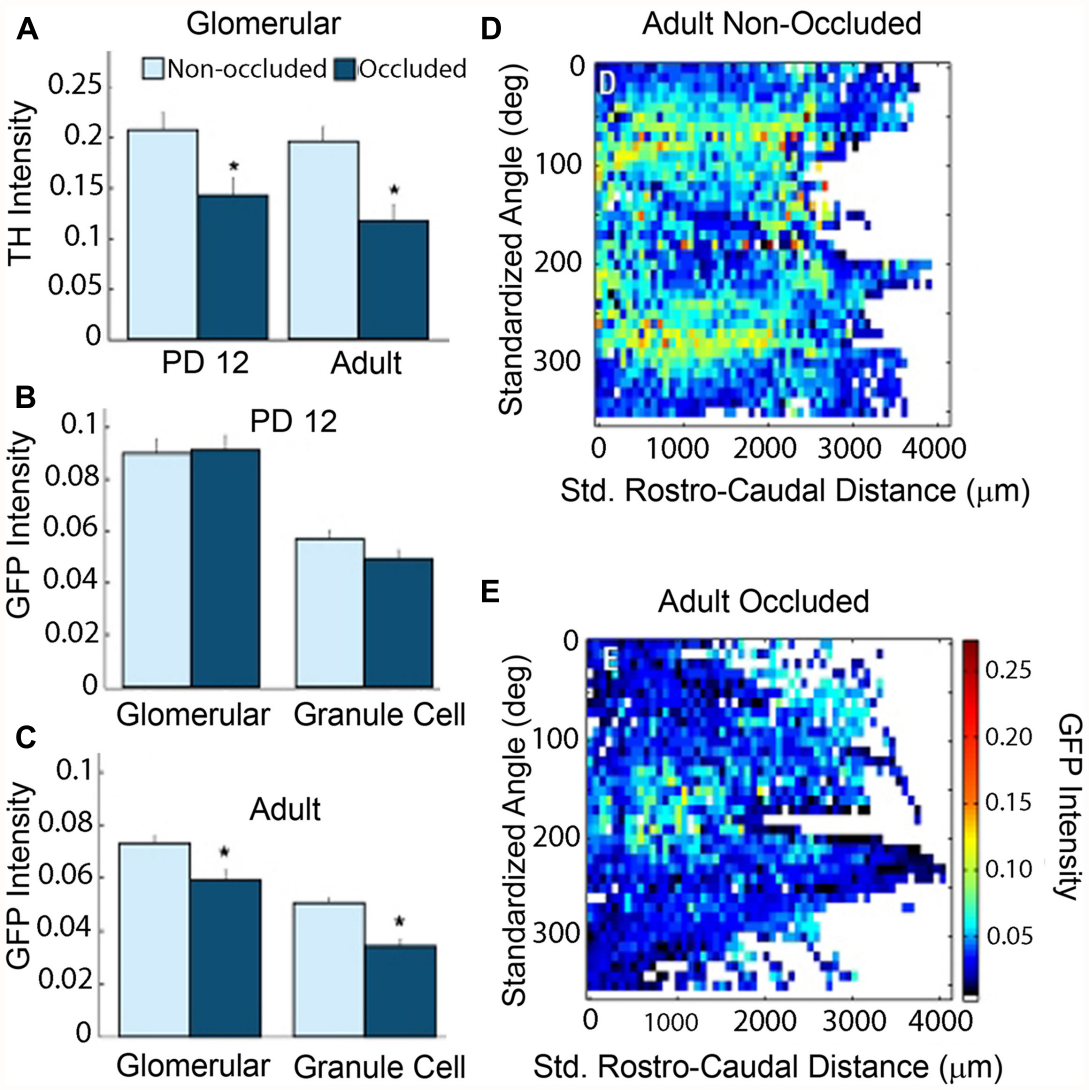
**Naris occlusion abolishes differential GFP staining pattern in adult animals.** Background subtracted intensities from 12-bit images were converted to a 0–1 scale and plotted. Details of image processing are given in Salcedo et al. (2011). **(A)** Significantly lower tyrosine hydroxylase (TH) intensity in occluded bulbs as compared to non-occluded bulbs confirmed that the occluded bulbs had reduced olfactory activity in both PD12 animals and adult animals (*t*-test, *p*-value = 1 × 10^-2^ and 3.6 × 10^-4^, respectively). **(B)** PD12 animals: mean GFP intensity did not vary significantly between occluded and non-occluded bulbs in either the glomerular or granule cell layers of PD12 bulbs. **(C)** Adult animals: mean GFP intensity fell significantly in both the glomerular and granule cell layers in the occluded bulbs of adult animals (*t*-test, *p*-values: 2.2 × 10^-3^ and 2.4 × 10^-8^, respectively). **(D–E)** Average intensity map in the occluded bulb **(E)** showed a markedly reduced differential patterning of GFP intensity throughout the GL as compared to the non-occluded bulb **(D)**. Data, with permission, from Salcedo et al. (2011).

Incoming fibers from the HDB show diffuse innervation across different layers of the bulb (Macrides et al., 1981; Zaborszky et al., 1986; Durand et al., 1998). This innervation is complete by postnatal day 12 (Salcedo et al., 2011). However, during further maturation, there is a distinct patterning of the innervation, with the predominant projections being directed to the glomerular layer (**Figures 2** and **3**) and sparser projections to other OB layers (Macrides et al., 1981; Salcedo et al., 2011). Within the glomerular layer, there are variations in projections (**Figure 2**) with some atypical glomeruli showing much denser innervation (Macrides et al., 1981; Gomez et al., 2005; Salcedo et al., 2011). The identity of odor inputs into these glomeruli, or the functional significance of their dense cholinergic innervation is, as yet, unclear. This suggests considerable pruning of cholinergic afferents during maturation (Salcedo et al., 2011).

Occluding sensory input to the bulb from one naris revealed that cholinergic input is modulated by olfactory activity (**Figure 3**). When unilateral naris occlusion was performed on postnatal day 2, the pattern and intensity remained unchanged up to postnatal day 12. However, significant reductions in intensities were observed in the ipsilateral bulb of the adult (**Figure 3**). In addition, the patterning was lost during this period. These results suggest that odor-induced activity is required for the maintenance and patterning of the cholinergic innervation.

## CHOLINERGIC RECEPTOR DISTRIBUTION IN THE OB

The anatomy and function of cholinergic receptor expression in the OB appear to be layer-specific. Quantitative autoradiography in rat OB slices point to the presence of presynaptic cholinergic terminals in the glomerular layer and in the EPL (Le Jeune et al., 1995), suggesting that the glomerulus and the secondary dendrites of MCs are important targets for cholinergic modulation. There is no evidence to suggest that cholinergic terminals form direct synaptic contacts on MCs. On the other hand, asymmetric cholinergic synapses have been described on dendrites of PG cells and GCs (Kasa et al., 1995). The prevalence of synaptic versus non-synaptic cholinergic signaling in the OB, like with other brain areas, remains unresolved to date.

The markers for the cholinergic receptors indicate an age-dependency of cholinergic receptor expression in the OB, with lowest levels of these markers observed at birth, and adult values observed by the end of 4–5 postnatal weeks (Le Jeune et al., 1996). This is consistent with the patterning of cholinergic innervation in the glomerular layer (Salcedo et al., 2011). The postnatal development of cholinergic innervation also extends to the EPL where GC dendrites make GABAergic contacts with the MCs.

Binding of [^125^I] α-bungarotoxin, a marker for the α7-containing nAChRs, was observed in the glomerular neuropil, suggesting a role for the α7 nAChR subtype in glomerular signaling. On the other hand, [^3^H] cytisine, which targets heteromeric nAChR subtypes, labeling α4β2*- and α3β4*-nAChRs (Xiao et al., 1998; Mao et al., 2008), binds to juxtaglomerular neurons and MCs. In addition to the ubiquitous α7 receptor, the rat OB also exhibits the presence of messenger RNA (mRNA) transcripts of nAChR genes that encode the α2, α3, α4, α5, α6, α9, β2, β3, and β4 subunits (Keiger and Walker, 2000), pointing to the possible expression of multiple receptor subtypes and, perhaps, indicating a diverse functional role of for nAChRs in the OB.

M1 and M2 mAChRs were shown to be highly expressed in the EPL indicating that mAChRs might be involved in regulating the dendrodendritic interactions between MCs and GCs. This has been experimentally verified by electrophysiological studies in acute slices (Castillo et al., 1999; Ghatpande et al., 2006; Pressler et al., 2007; Ghatpande and Gelperin, 2009), as well as *in vivo* (Tsuno et al., 2008).

## CHOLINERGIC SIGNALING IN THE OB

**FIGURE 4:**
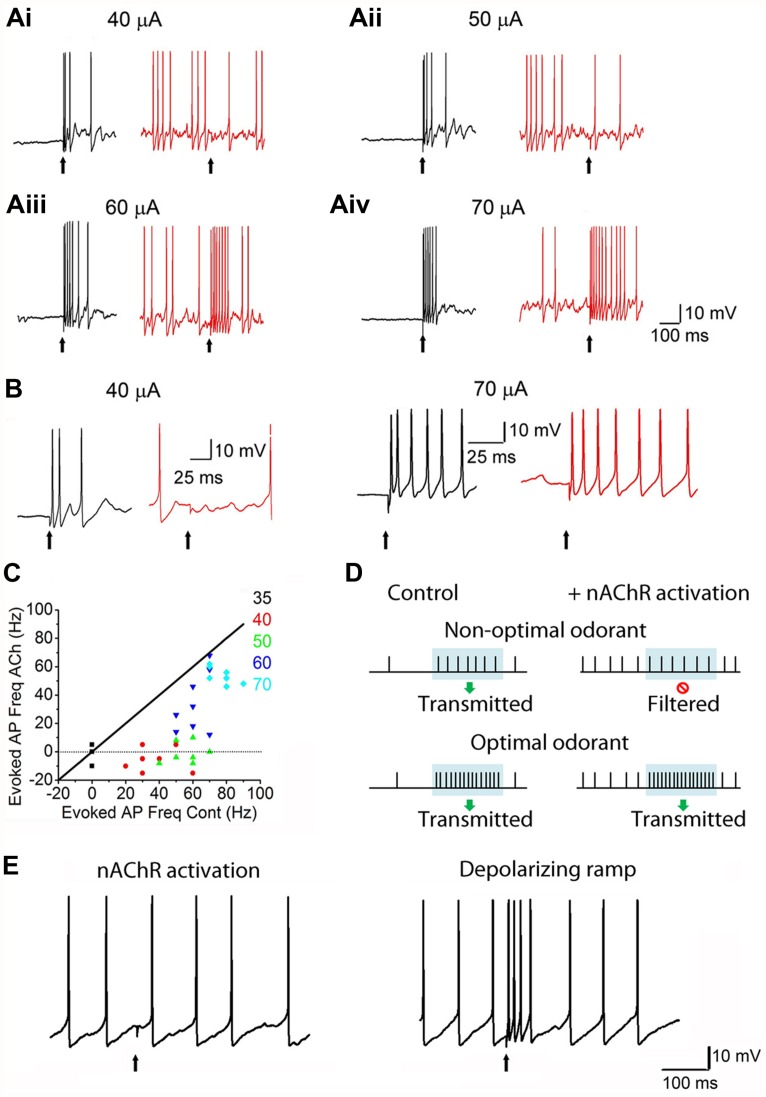
**nAChRs act as high-pass filters of glomerular output. (Ai)**: Responses of MCs to ON stimulation, recorded under current-clamp. Left: **(A)** 40 μA ON stimulus causes an MC to exhibit a burst of spikes. Right: during a ACh/At-mediated depolarization (and enhanced background firing), the 40 μA stimulus fails to evoke a response in the same MC. ACh/At refers to a 1 s focal application of 1 mM ACh in the presence of 2 μM atropine, the latter also added in the bath in order to block mAChRs. **(Aii–iv)**: Similar data for 50, 60, and 70 μA stimuli, respectively. While the 50 μA stimulus also fails to evoke a response during the ACh/At-mediated spiking in the same MC as in Ai, the MC responds to higher stimuli with increased spiking. In all cases, control traces are in black and traces in the presence of ACh/At are in red. **(B)**: Expanded traces from Ai and Aiv. **(C)**: Scatter plot of net increase in spiking upon ON stimulation, during the ACh/At-mediated depolarization, plotted against the same during control conditions. Data is from the same cell as in **(A,B)**. Net increase in spiking was calculated by subtracting the mean firing frequency before ON stimulation from the mean firing frequency during the 100 ms window after ON stimulation. While responses to all stimulus intensities were suppressed during the ACh-mediated depolarization, lower intensity stimuli (up to 50 μA) show a filtering of MC responses (not different from 0). Diagonal line (slope = 1) is where the points would lie if there were no ACh-mediated filtering. **(D)**: Cartoon summarizing the effects of nAChRs on MC responses. Period of odor exposure shown by shaded box. Under non-optimal conditions (weak odor), a MC fires a burst of APs during odor exposure leading to signal transmission. On the other hand, nAChR activation, causes an increase in basal MC firing but shows no net change in firing patterns during the period of odor exposure thus resulting in filtering of the response. Under optimal conditions (i.e., strong odor), there is a net increase in MC firing during odor exposure both under control conditions and when nAChRs are activated. **(E)**: Filtering shown in the presence of ACh/At (left trace) is not seen when the same cell is depolarized via current injection to elicit APs in the absence of ACh/At (right trace, from the same cell). This suggests that optimal excitation-driven feedback inhibition requires the activation of more than one MC, and that the filtering is not merely a result of MC membrane depolarization. Figures **(A–C,E)** adapted, with permission, from D’Souza and Vijayaraghavan (2012).

A major site for nAChR regulation is the glomerulus of the OB. Consistent with autoradiographic studies (Le Jeune and Jourdan, 1993; Le Jeune et al., 1995) functional nAChRs have been described in MCs and ET cells (D’Souza and Vijayaraghavan, 2012; D’Souza et al., 2013). These functional receptors belong to the heteromeric α3β4*-nAChR and the α4β2*-nAChR subtypes. On MCs, nAChRs appear to be selectively clustered at the primary dendritic tuft within the glomerular neuropil. Removing the primary dendrite drastically attenuates ACh-induced nAChR currents (D’Souza and Vijayaraghavan, 2012). Overall, these results suggest that nAChRs are expressed primarily on excitatory neurons in the glomerular microcircuit. Further, activation of glomerular nAChRs leads to increased glutamate release within the neuropil, resulting in an excitation-dependent feedback inhibition onto the MCs and ET cells. This occurs via increased GABA release from activated juxtaglomerular interneurons, presumably the PG cells (D’Souza and Vijayaraghavan, 2012; D’Souza et al., 2013). The predominant effect of nAChR activation appears to be to inhibit incoming signals from the ORNs, leading to a “filtering” mechanism wherein only ORNs excited above a certain intensity threshold transmit their information to cortex (**Figure 4**). A possible mechanism for this inhibition is the shunting of ORN inputs due to the increase in the membrane conductance of MCs upon the opening of a large number of channels, particularly the nAChRs and GABA receptors. Thus, the receptors act as high pass filters that attenuate weak signals while allowing stronger ones to pass, thus setting odor detection thresholds.

Potentially important players in the increased GABA release within the glomerulus upon nAChR activation are the ET cells (D’Souza et al., 2013), a population of OB neurons whose physiological properties have been characterized over the last decade. ET cells are thought to be a major source of excitation for juxtaglomerular neurons (Hayar et al., 2004), as well as drivers of feed-forward MC excitation via glutamate release within the glomerulus (De Saint et al., 2009; Najac et al., 2011; Gire et al., 2012). As targets for neuromodulation by cholinergic (D’Souza et al., 2013), serotonergic (Liu et al., 2012) and endocannabinoid receptor-mediated (Wang et al., 2012) mechanisms, ET cells are well placed to play a vital role in the state-dependent control of OB function. Similar to MCs, glomerular nAChR activation leads to an enhancement of ET cell excitability. This excitation, along with MC excitation, is likely responsible for the increase in the frequency of GABA release within the glomerulus upon nAChR stimulation. There is one report suggesting that a subpopulation of PG cells might, themselves, express nAChRs (Castillo et al., 1999) but their contribution to the glomerular microcircuit is yet to be determined.

**FIGURE 5:**
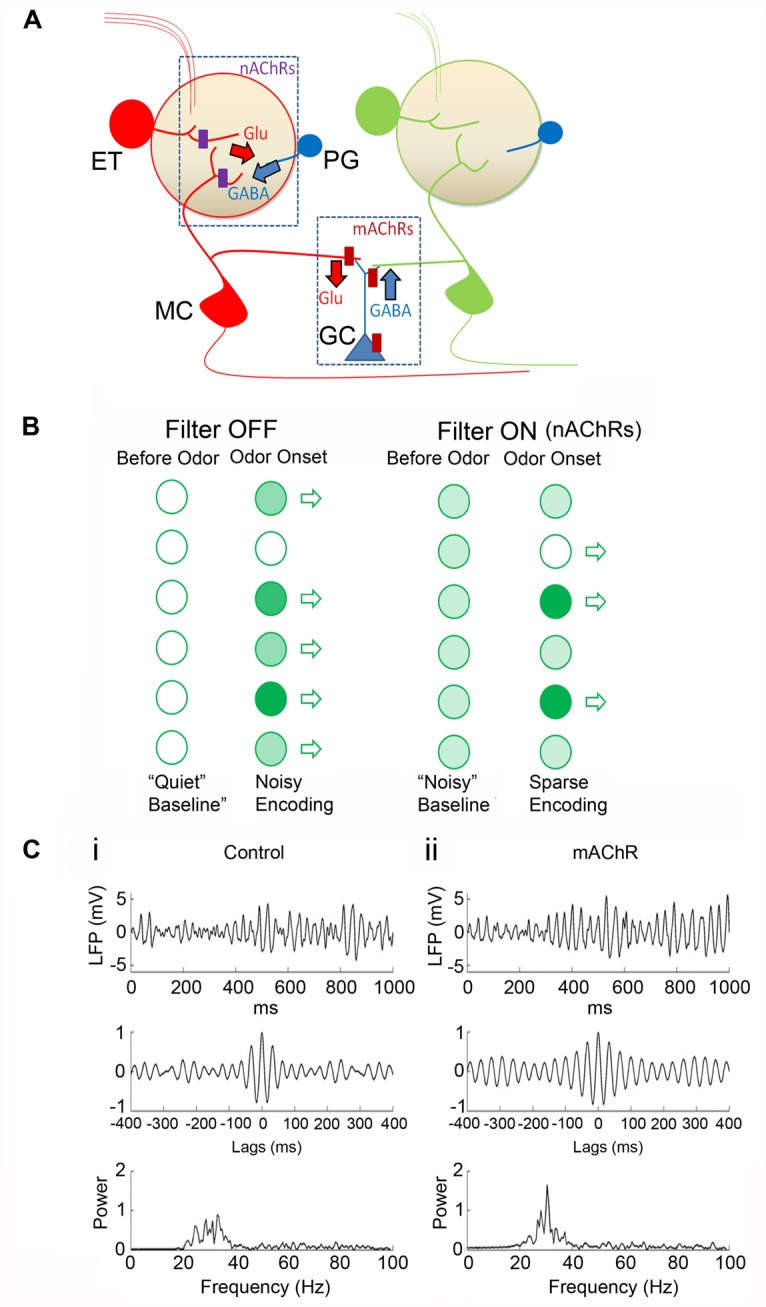
**A working model for cholinergic modulation of OB circuitry. (A)** Cartoon of the OB circuit showing major sites of cholinergic inhibition. In the glomerular microcircuit, nAChRs (purple rectangles) are expressed in the primary dendritic tufts of MCs and on the ET cell (in red). Activation of these receptors depolarizes the neurons resulting in the release of glutamate (Glu). Released glutamate excites nearby PG cells (blue) eliciting a feedback GABA release on to the excitatory neurons. **(A)** Similar feedback circuit exists at the dendrodendritic synapses between GCs and secondary dendrites of MCs allowing for lateral inhibition of adjacent MC dendrites. **(B)** Cartoon showing glomerular output (open arrows). In the absence of receptor activation (Filter OFF) there is less baseline activity (“Quiet baseline.”) Odor stimulations at different strengths (light and dark green circles for weak and strong stimulation, respectively) are transmitted through. Upon nAChR activation, (Filter ON), excitation of ET cells and MCs lead to a noisier baseline (i.e., all light green). However, upon odor input, excitation-driven inhibition results in filtering out of weaker inputs, such that only strong ones pass through. Further, increased basal activity also allows for potential “inhibitory readouts” (open circle) where net MC firing rates can be reduced to levels below that prior to odor onset (see **Figure 4C**). The time window of the nAChR-evoked inhibition will determine the efficacy of this filter. Thus determining the temporal patterns of ACh release, in relation to behavioral stages, is necessary in order to predict the direct consequences of this filter mechanism. **(C)** Modeling the activation of mAChRs in the OB (with permission from Li and Cleland, 2013). In this model, mAChR activation does not alter MC firing rates but the receptor activation enhances sLFP oscillatory power and imposes more stringent phase locking between MC spikes and sLFP oscillations. **(i)** Control responses: simulated sLFP during odor presentation **(top)** with autocorrelation **(middle)** and power spectrum **(bottom)**. **(ii)** Same as **(i)** but during active mAChR modulation. In response to odor, MC spikes were locked to the gamma frequencies under both control conditions and upon mAChR activation, but the responses were more tightly phase constrained when mAChRs were active. This is consistent with the idea that modulation of lateral inhibition by mAChR signaling at GC-MC synapses imposes a stronger synchronization of MC firing in the OB.

mAChRs, on the other hand, appear to mainly control a second inhibitory circuit in the OB, involving GCs and the lateral dendrites of MCs (see **Figure 5A**) within the EPL. Activation of M1-mAChRs, via mobilization of endoplasmic reticulum store calcium, release GABA onto the MCs at the dendrodendritic synapses between GCs and MCs (Castillo et al., 1999; Ghatpande et al., 2006; Ghatpande and Gelperin, 2009). At the same time, M1-mAChRs increase GC excitation thus providing an additional inhibitory drive on to MCs (Pressler et al., 2007). Similar mechanisms of cholinergic modulation were also observed in the accessory OB where M1-like mAChRs control GC-to-MC inhibition, while nAChR activation increases MC excitability (Smith and Araneda, 2010).

## mAChRs AND nAChRs CONTROL OB FUNCTION VIA DISTINCT MECHANISMS

Results from a variety of behavioral and *in vivo* electrophysiological studies point to the importance of cholinergic receptor activation in modulating the detection and discrimination of odors, as well as in olfactory perceptual learning, i.e., learning to distinguish between two or more perceptually similar odors (Fletcher and Wilson, 2002; Wilson and Stevenson, 2003; Wilson et al., 2004; Fletcher and Chen, 2010). For instance, increasing the level of ACh in the OB results in the sharpening of the molecular “receptive field” of individual MCs in response to odors, while the addition of nAChR and mAChR blockers into the OB impairs the ability to distinguish between similar odors, both at the level of MC spike frequency, as well as in behavioral tests (Chaudhury et al., 2009).

In addition to modulating odor detection and discrimination (Chaudhury et al., 2009), nAChRs are also involved in olfactory working memory. Mice that lacked the α7 nAChR showed impairments in working memory when compared to wild type mice (Young et al., 2007a), while acute nicotine administration could fully restore deficits in olfactory working memory in a transgenic mouse model that overexpressed the caspase-3 protein (Young et al., 2007b). Olfactory working memory could also be enhanced in rats via subcutaneous administration of specific agonists for the α4β2 and the α7 receptor subtypes (Rushforth et al., 2010).

mAChRs have been shown to be involved in the behavioral state-dependent control of dendrodendritic synapses between MCs and GCs (Tsuno et al., 2008). Results from this work indicated that inhibition of MCs by GCs was most enhanced during the slow-wave sleep state and successively weaker during light sleep, awake immobility, and awake moving states. Activation of mAChRs were responsible for this inhibition. This supports the observation that ACh levels in the brain are higher in the awake state than when the animal is asleep. Real time monitoring of ACh levels demonstrate that the awake state is characterized by low levels of ACh (Parikh and Sarter, 2008), though relatively higher than that during slow wave sleep, which might signal via mAChRs to maintain a tonic GABAergic control on the basal firing rates of MCs. It appears, therefore, that an important function of cholinergic input to the OB is to inhibit the activity of GCs, thereby disinhibiting the MCs during wakefulness and behavior. *In vivo*, mAChRs have been suggested to play a role in potentiating the firing rates of MCs upon stimulation of the basal forebrain (Zhan et al., 2013), as well in olfactory perception and short-term olfactory memory (Chaudhury et al., 2009; Devore et al., 2012).

Interestingly, optogenetic excitation of cholinergic neurons in the HDB of anesthetized animals inhibits the basal firing rate of (M/T) cells, while also inhibiting the basal firing of the GABAergic granule and PG cells (Ma and Luo, 2012). This observation is quite surprising because, as described above, MCs have been shown to be excited by nAChR activation. Further, work from a number of labs using acute OB slices have demonstrated that mAChRs, in contrast to the optogenetic study, excite GCs, leading to increased GABAergic postsynaptic currents in MCs (Pressler et al., 2007). It is therefore unclear as to how cholinergic input inhibits GCs *in vivo*. It must be pointed out, however, that general anesthetics have effects on nAChR function and might, therefore, confound interpretations when testing cholinergic effects in anesthetized animals (Hara and Harris, 2002; Weber et al., 2005; Liu et al., 2009). Further, a more recent study demonstrated that exciting the cholinergic axons in the OB, instead of exciting the cell bodies in the HDB, leads to an enhancement of M/T cell firing (Rothermel et al., 2014). This observation suggests that activating cholinergic somata in the HDB may lead to indirect inhibition of M/T cells and other bulbar neurons, via pathways that remain to be elucidated (Rothermel et al., 2014).

Activation of basal forebrain cholinergic neurons also results in the sharpening of M/T cell responses so that when these inputs are activated, M/T cell responses to the optimal odorant (i.e., an odorant that elicits the maximal response in the M/T cell under control conditions) are enhanced, while responses to non-optimal odorants are suppressed (Ma and Luo, 2012). It should be noted, however, that direct activation of the cholinergic axons in the OB did not lead to such a suppression for non-optimal odors; instead, excitation of these fibers led to an enhancement of odor-evoked M/T cell responses independent of control response strengths (Rothermel et al., 2014). Thus, there appears to be qualitative differences between activating cholinergic cell bodies within the HDB and activating their fibers in the OB to study the effects of cholinergic input on bulbar function. Regardless of these differences, these results strongly imply that cholinergic input to the OB is responsible for enhanced olfactory function, potentially playing a central role in the detection of weak odors and in the discrimination of chemically similar odorants. Computational models based on experimental observations suggest that mAChRs are likely responsible for the generation of gamma oscillations in the OB while the activation of nAChRs sharpen the tuning curves of MCs in response to odor input (Li and Cleland, 2013), thus pointing to a role of mAChRs (via the modulation GC-MC interactions in the EPL) in controlling MC spike timing, and the role of nAChRs in enhancing contrast between activated glomeruli.

## OTHER PLAYERS IN THE CHOLINERGIC MODULATION OF THE OB

Our knowledge of cholinergic modulation of the OB output is far from complete as we are still discovering the extent of receptor distribution and cell types that they can act upon. The functional role that the α7 receptor plays in modulating OB function is still unresolved. Anatomical studies have revealed that α7 nAChRs are highly expressed in the glomerulus (Le Jeune et al., 1995; Hellier et al., 2010), while behavioral studies point to an important role for this receptor subtype in olfactory function (Hellier et al., 2010, 2012). However, electrophysiological studies suggest that α7 nAChRs do not play a significant role in modulating the spontaneous activity of MCs (D’Souza and Vijayaraghavan, 2012). It is possible that glomerular α7 nAChRs are expressed not on the glomerular tufts of MCs, but on the axon terminals of ORNs, which provide the input to the OB. Other possibilities include the expression of these receptors on glomerular astrocytes, or on centrifugal fibers that innervate the glomerulus. The observation that the release of other neuromodulators such as serotonin and noradrenaline can be altered by cholinergic activation (Decker and McGaugh, 1991; Levin and Simon, 1998), and that circuits in the OB are also modulated by these two neuromodulators (Fletcher and Chen, 2010; Devore and Linster, 2012; Liu et al., 2012), point to the possibility of a sophisticated interplay between these three neuromodulatory systems in regulating the output of the OB. While α7 nAChRs do not appear to play a significant role in inducing nicotinic currents in MCs, or altering the frequency of spontaneous postsynaptic currents on them, they might play a role in mediating plasticity in the OB. This is supported by observations that the receptor is important for olfactory learning (Hellier et al., 2010, 2012; Rushforth et al., 2010).

Similarly, we have no information on the role M2-mAChRs play in the cholinergic modulation of the bulbar output. Anatomical evidence suggests that these receptors are localized on GC synapses in the EPL, on second-order GABAergic neurons in the infra-mitral cell layer, and on some juxtaglomerular GABAergic interneurons, suggesting complex inhibition/disinhibition roles for these receptors on MC output (Crespo et al., 2000).

Our knowledge of the function and regulation of various juxtaglomerular interneurons is incomplete as well. For example, the short axon cells, a type of juxtaglomerular cells that mediates interglomerular inhibition (Aungst et al., 2003), have been studied with increased detail only in recent years (Kiyokage et al., 2010; Liu et al., 2013; Whitesell et al., 2013). Their role in cholinergic modulation remains unresolved, although it’s possible that this cell type was previously identified as “bipolar PG cells” that exhibited prominent, slow, inward currents upon nicotinic activation (Castillo et al., 1999).

## POSSIBLE MECHANISMS FOR CHOLINERGIC CONTROL OF OLFACTORY CODING

The vast repertoire of cholinergic receptor subtypes expressed throughout the brain exhibit a variety of physiological properties. These include the sensitivity of these receptors to ACh, as well as their desensitization rates. A major puzzle in the field of cholinergic function is to understand the roles played by these different receptor subtypes. Cholinergic transmission in the brain can be broadly classified as occurring via two modes, synaptic, and diffusion-based, resulting in the release of ACh with concentrations that vary over orders of magnitude. Varying concentrations of ACh acting on receptor subtypes exhibiting a myriad of sensitivity and desensitizing properties indicate a dynamic control of sensory processing over multiple timescales that is dependent on the behavioral state of the animal.

As we described earlier, behavioral and *in vivo* work have shown that during light sleep or awake immobility, a low, tonic level of ACh primarily activate mAChRs expressed on GCs (Tsuno et al., 2008). This sets a basal cholinergic tone for GABAergic control of OB output. In contrast, during the anticipational/attentional phase of behavior, there is a rapid and transient increase in MC firing, and it has been suggested that this spontaneous activity in the alert animals might be driven by basal forebrain cholinergic activity (Rinberg and Gelperin, 2006). Consistent with this finding, real-time measurements of ACh levels in the brains of rats performing attention-dependent tasks indicate that cholinergic activity acts on three distinct timescales depending on effort: (1) cue-evoked transient increases in ACh levels that act on the scale of seconds, (2) pre-cue cholinergic signals on the scale of tens of seconds when the rat is anticipating or predicting a cue, and (3) a tonic level of activity that lasts for minutes throughout the session (Parikh et al., 2007, 2008). The transient increase in ACh levels during sustained attention would be sufficient to activate the lower affinity nAChRs, especially the slowly desensitizing heteromeric receptor subtypes. This excitation, in conjunction with feedback GABAergic inhibition, could potentially result in the gating of odor input so that only MCs belonging to strongly activated glomeruli are excited. Such a mechanism would potentially filter out “noise” from weakly activated glomeruli, and lead to enhanced contrast between odor maps encoding chemically similar odors. Noise, in this context, refers to the non-optimal activation of glomeruli via weakly excited ORNs (see **Figure 5B**). This model, therefore, predicts a role for both tonic and phasic modulation for cholinergic inputs (Parikh and Sarter, 2008; Sarter et al., 2009a,b).

Direct excitation of MCs by cholinergic activation has important implications for odor processing. First, the depolarization of MCs can drive them to spike with a high basal firing rate (D’Souza and Vijayaraghavan, 2012). If attention-dependent cholinergic input leads to an increase in the basal firing rate of MCs, it would allow an odor input to alter the frequency, as well as the timing, of spikes. For instance, a decrease in spike frequency or changes in the fine temporal structure at the level of individual action potentials, upon odor input, would not be possible if the cells were not already firing in the first place. Having a baseline firing rate before odor input therefore provides a template for the incoming odor input to manipulate and provide more information to process. Second, depolarization of MCs *before* the onset of an odor signal would trigger the PG-cell driven feedback inhibition, such that, only MCs belonging to glomeruli that receive a strong odor input would transmit the information to the cortex. Third, increasing the basal firing frequency of MCs would increase the probability of coincident synaptic excitation of GCs. Since GCs form reciprocal dendrodendritic contacts with the lateral dendrites of MCs, an increase in the excitation of GCs would, in turn, increase lateral inhibition between MCs (Arevian et al., 2008). Slice and computational studies have implicated the role of GC-mediated lateral inhibition in the synchronization of MC action potentials (Galan et al., 2006; Schoppa, 2006). The cause of this synchrony is the near-simultaneous recovery of MCs from synchronized GABAergic inhibition, and is thought to underlie synchronous neuronal oscillations in the gamma frequency (Schoppa, 2006). Computational modeling supports the idea that cholinergic excitation of the OB circuit increases the synchronization, as well as the sparseness, of MC action potentials in response to odor input (de Almeida et al., 2013). The observations that cholinergic influence mediate gamma frequency oscillations within neuronal populations (Dickson et al., 2000; Simon et al., 2011) suggest a possibility for a synchronized activity baseline in the OB prior to odor input that could be altered by a subsequent inhalation of odors, providing for a mechanism for allowing the detection and higher-order processing of olfactory information.

## OB CHOLINERGIC MODULATION AND NEURODEGENERATIVE DISEASES

The notion that olfactory dysfunction is one of the early symptoms in neurodegenerative diseases is gaining recognition (Attems et al., 2014). As olfactory deficits have been shown to manifest themselves years prior to onset of characteristic symptoms, they might act as early biomarkers of these diseases (Barresi et al., 2012). Major deficits in odor detection, identification, and discrimination have been described in Parkinson’s disease (PD) patients, prior to the onset of motor disturbances (Mesholam et al., 1998; Tissingh et al., 2001; Doty, 2009), even leading to a theory that PD might be a primary olfactory disorder (Hawkes et al., 1999).

Similar evidence exists for patients with Alzheimer’s disease (AD) where early loss of olfactory discrimination and anosmia has been reported (Christen-Zaech et al., 2003; Djordjevic et al., 2008). Changes in the number of dopaminergic PG cells and loss of OB volume have been described in AD (Mundinano et al., 2011). In mouse models of AD, early onset of olfactory deficits also corresponds to early depositions of amyloid β protein prior to central pathology (Wesson et al., 2010; Kim et al., 2011).

The cholinergic hypothesis for diseases like AD has had dominance for many decades (Bartus et al., 1982; Coyle et al., 1983; Bartus, 2000) and has led to the development of the only approved drugs for the treatment for early and mild dementia. While cholinergic dysfunction is likely to be one of many causes for neurodegeneration (Craig et al., 2011), these studies nonetheless suggest a dominant role for this neurotransmitter system. Studies with patients suffering from Parkinson’s disease have indicated that olfactory deficits, seen early in the disease process, correlates with cholinergic degeneration rather than the nigro-striatal dopaminergic neuron deficits (Bohnen et al., 2010), once again confirming the correlation between olfactory function and the cholinergic system.

Our studies indicate that distribution of cholinergic fibers in the OB is intricately connected to olfactory sensory input (**Figure 3**). Unilateral naris occlusion results in a loss of pruning of incoming cholinergic fibers in the adult and results in diffuse innervation of the OB similar to that seen in young (day 12) animals (Salcedo et al., 2011). Does disruption of axonal pruning in the bulb alter the survival of HDB cholinergic neurons? We do not know this, but if loss of axons results in “die-back” and delayed death of neuronal soma in the basal forebrain, it is possible to conceptualize a mechanism that connects sensory environment to neurodegeneration and memory loss observed in AD or other diseases.

## CONCLUSION

In most mammals, the ability to discriminate, effectively, benign odors from those that could signal danger is an essential prerequisite for survival. It is, therefore, logical that systems signaling arousal and attention are brought to bear during tasks of odor discrimination. The key transmitter system invoked in these olfactory tasks involves the cholinergic projections from the basal forebrain, long thought to be involved in attention, arousal, learning, and memory. In the olfactory system, it is well accepted that significant processing of odor information occurs at the OB.

A simple model, based on current state of our knowledge, would postulate that nAChR activity dominates at the glomerular microcircuit, while mAChRs control the GC-driven modulation of MC firing (**Figure 5**). The key process in cholinergic modulation of OB functions, appears to be GABAergic signaling. Activation of nAChRs drives glomerular inhibition via the indirect excitation of PG cells. This allows for normalization of glomerular excitation, setting thresholds for transfer of information. The excitation of MCs and ET cells by nAChRs also increase baseline firing, potentially providing a template for net negative readouts in firing frequencies, as well (see **Figures 4C** and **5B**).

At the same time mAChR-driven excitation of GCs and their modulation of GABAergic signaling at the GC-MC dendrodendritic synapses allows for lateral inhibition. Recovery from inhibition across MCs aids in synchronizing firing which is thought to facilitate the integration of incoming information at a population level (**Figure 5C**). Ongoing efforts at further localizing the relevant receptors and at manipulating cholinergic inputs in awake animals performing olfactory tasks will shed more light on this important modulation of a sensory modality by cholinergic processes.

## Conflict of Interest Statement

The authors declare that the research was conducted in the absence of any commercial or financial relationships that could be construed as a potential conflict of interest.

## ABBREVIATIONS

ACh: acetylcholine
ET cell: external tufted cell
HDB: horizontal limb of the diagonal band of Broca
M/T cells: mitral and/or tufted cells
mAChR: muscarinic acetylcholine receptor
MC: mitral cell
nAChR: nicotinic acetylcholine receptor
OB: olfactory bulb
ON: olfactory nerve
ORN: olfactory receptor neuron
PG cell: periglomerular cell
GC: Granule cells.
